# Comparisons between the 35 mm Quadrature Surface Resonator at 300 K and the 40 mm High-Temperature Superconducting Surface Resonator at 77 K in a 3T MRI Imager

**DOI:** 10.1371/journal.pone.0118892

**Published:** 2015-03-26

**Authors:** Manli Song, Jyh-Horng Chen, Ji Chen, In-Tsang Lin

**Affiliations:** 1 State Key Laboratory of Molecular Vaccinology and Molecular Diagnostics, School of Public Health, Xiamen University, Xiamen Fujian, China; 2 Center for Molecular Imaging and Translational Medicine, School of Public Health, Xiamen University, Xiamen Fujian, China; 3 Interdisciplinary MRI/MRS Lab, Department of Electrical Engineering, National Taiwan University, Taipei, Taiwan; 4 Graduate Institute of Biomedical Electronics and Bioinformatics, National Taiwan University, Taipei, Taiwan; 5 Department of Physiology and Neurobiology, Medical College of Xiamen University, Xiamen Fujian, China; Texas A&M University, UNITED STATES

## Abstract

This study attempts to compare the signal-to-noise ratio (SNR) of the 40 mm High-Temperature Superconducting (HTS) surface resonator at 77 K and the 35 mm commercial quadrature (QD) surface resonator at 300 K in a 3 Tesla (T) MRI imager. To aquire images for the comparison, we implemented a phantom experiment using the 40 mm diameter Bi_2_Sr_2_Ca_2_Cu_3_O_x_ (Bi-2223) HTS surface resonator, the 35 mm commercial QD surface resonator and the 40 mm professionally-made copper surface resonator. The HTS surface resonator at 77 K provided a 1.43-fold SNR gain over the QD surface resonator at 300 K and provided a 3.84-fold SNR gain over the professionally-made copper surface resonator at 300 K on phantom images. The results agree with the predictions, and the difference between the predicted SNR gains and measured SNR gains is 1%. Although the geometry of the HTS surface resonator is different from the QD surface resonator, its SNR is still higher. The results demonstrate that a higher image quality can be obtained with the HTS surface resonator at 77 K. With the HTS surface resonator, the SNR can be improved, suggesting that the HTS surface resonator is a potentially helpful diagnostic tool for MRI imaging in various applications.

## Introduction

As the development in biomedicine, psychology, and clinical progresses both higher spatial and temporal resolutions are needed to observe the finer details of certain structures. However, as the imaging resolution goes higher, signal-noise-ratio (SNR) will become the limiting factor for accurate quatitative magnetic resonance (MR) microscopy. Therefore, improving the SNR is essential for elevating image quality. High-temperature superconducting (HTS) radio-frequency (RF) resonators have been regarded as a promising tool for MR imaging due to their low impedance of below critical temperature and are currently used to improve the sensitivity of RF detecting resonators. The development of relatively affordable bismuth based HTS tapes, such as Bi_2_Sr_2_Ca_2_Cu_2_O_3_ (Bi-2223) and Bi-2212 tapes, have become one of researchers' potential choices for RF resonators in MRI. Grasso et al. [1] measured the quality factors (QF) of surface resonators made with Bi-2223 tapes and observed that they were only slightly less than that of YBa_2_Cu_3_O_y_ (YBCO) films. Cheng et al. [2] built a 5-inch tape RF receiving resonator for a 0.21 Tesla (T) MRI system, and demonstrated a 3-fold SNR improvement over an equivalent room temperature copper resonator, which indicates the feasibility of HTS tapes for MRI. In the previous study, we demonstrated HTS tape resonators of different sizes in a 3T MRI [3,4,5]. However, the versatility of HTS RF surface resonator has not been widely compared with any kind of commercial surface resonators. Thus the 35 mm quadrature (QD) surface resonator (Rapid Biomedical Corp., Wurzburg, Germany) was chosen for this comparision in this study.

It has also indicated that the samples are frozen [3,6] when the temperature drops to the 77 K. The image of the frozen sample then becomes dark due to the loss of water. The loss of water further diminishes the SNR. The previous cryostat led to an unsuccessful outcome in animal experiments due to incomplete thermal insulation. Thus we have also designed a temperature stable cryo-system [7]. It is difficult to design due to its thermal insulation requirements. Glass is used because it can be a unibody that provides sufficient vacuum space for preventing thermal convection, and it is cheaper and easier to use compared with fiberglass composites (G10) [7]. The limited autonomy can be improved by supplying a liquid nitrogen (LN2) stream through flexible pipes connected to a standard remote cryostat.

The QD surface resonator was used to obtained rat brain images and conduct rat fMRI studies [8]. The rat brain images of the QD surface resonator had to be clear to be analyzed, and the scanning time was about 1 hour with our 3T MRI. Here an 0.5 S.m-1 NaCl-solution phantom experiment was used to evaluate the 40 mm diameter Bi-2223 HTS surface resonator and compared it with the QD surface resonator. First of all, in the phantom images the HTS surface resonator at 77 K provided a 1.43-fold SNR gain over the QD surface resonator at 300 K and also provided a 3.84-fold SNR gain over the professionally-made copper surface resonator at 300 K. From the phantom imaging results, we notice that the HTS surface resonator at 77K shows a better image quality, and the SNR is much higher comparing with the QD surface resonator at 300K. This is also our first report on comparisons between the 35 mm commercial QD surface resonator and the 40 mm high-temperature superconducting surface resonator in our 3T MRI.

## Materials and Methods

### Hardware

**Fig 1:**
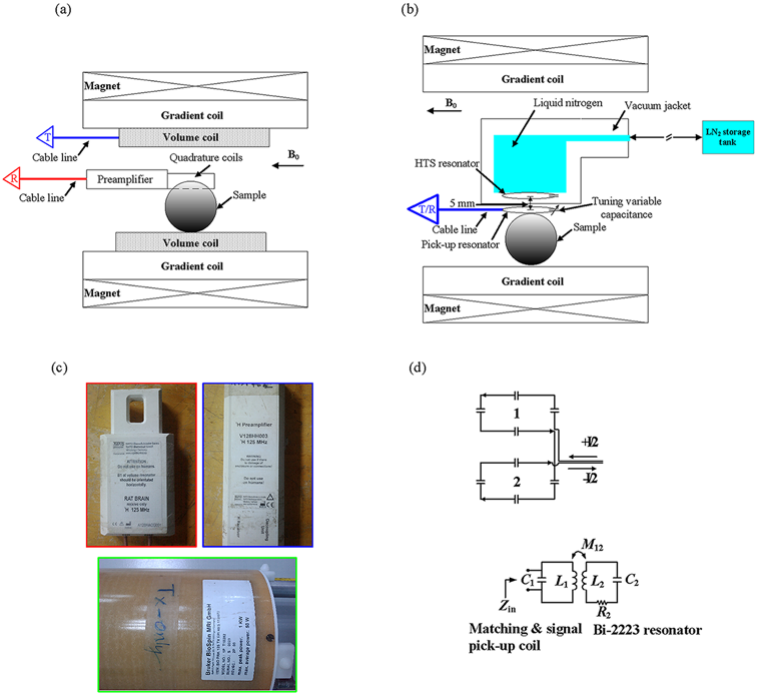
The experimental setup of the different coils and the details of the QD surface resonator. (a) The 3T system setup of the phantom experiment, where the QD surface resonator was put inside the QD volume resonator. (b) The 3T system setup of the phantom experiment, where the matching and signal pick-up resonator with a tuning variable capacitance was put under the cryostat and the HTS surface resonator. (c) Top left: the QD surface resonator, Top right: the preamplifier, and the bottom: the QD volume resonator. (d) The top: The geometry of QD form circuit, the bottom: the circuit diagram of the matching and signal pick-up resonator with the variable tuning trimmer capacitor C_1_ and the Bi-2223 surface resonator.

MR experiments were performed on a 3T MRI system (Bruker BioSpec, Karlshruhe, Germany) with an inserted gradient, of which the maximum gradient strength was 200 mT/m with an inner diameter of 12 cm. Fig. 1 (a) shows the experimental setup of the commercial coil, where the 35 mm QD surface resonator (Rapid Biomedical Corp., Wurzburg, Germany) and the phantom were both surrounded by the QD volume resonator (Bruker Corp., Karlsruhe, Germany). And, the experimental setup of the HTS/copper surface resonators which were placed inside the cryostat were shown in Fig. 1 (b), where the matching and signal pick-up resonator with a tuning variable capacitance were positioned between the HTS/copper surface resonator and the phantom. In a traditional scan setup, the radiofrequency pulses are transmitted by the QD volume resonator and received by the 35 mm QD surface resonator, as shown in Fig. 1 (a). The module of the transmitted resonator is BIOPRK125TXEPIRES112/072 and the outer/inner diameter is 112/072 mm (the bottom of Fig. 1 (c)), while the module of the QD surface resonator is A125HACG001 and the diameter is 35 mm (the top left of Fig. 1 (c)). The QD surface resonator is in a saddle shape QD form (the top of Fig. 1 (d)) and the QD surface resonator was connected the preamplifier V128HH003 (the top right of Fig. 1 (c)). The details of these resonators are listed in Table 1.

**Table 1:** General specification of the HTS, copper, QD surface and QD volume resonators.

	The HTSsurface resonator	The copper surface resonator	The QD surface resonator	The QD volume resonator
**Material**	Bi-2223 tape	Copper	Copper	Copper
**Geometry**	Single loop form and diameter 40 mm	Single loop form and diameter 40 mm	Quadrature form and diameter 35 mm	Quadrature form and inner diameter 72 mm outer diameter 112 mm
**Configuration**	Transceiver	Transceiver	Receiver only	Transmitter only

The experiment was setup so that the radiofrequency pulses were transmitted and received by the HTS/copper surface resonators which were placed inside the cryostat, as shown in Fig. 1 (b). The HTS tape was soldered to a high-Q_s_ chip capacitor (22 pF and Q ≈ 1000 at 125 MHz, American Technical Ceramics, NY, USA) to form a resonator and bended into a circular shape with a diameter of 4 cm. The material used for the HTS tape was Bi-2223 (Innova Corp., Beijing, China). The minimum bending radius of the HTS tapes is 20 mm due to the limitation of radius of curvature. And the HTS tapes is easy to be broken when the diameter is less than 40 mm. Therefore, to maintain the superconducting properties of the HTS tapes, an 40 mm HTS surface resonator was used in this study. The wires with a multi-filamentary structure showed a critical temperature of 110 K and an engineering critical current density greater than 9000 A/cm^2^ at 77K. The thickness and width of the raw Bi-2223 tape were 0.23 mm and 4.1 mm, respectively, with a 10μm thick tin alloy sheath to provide mechanical support for the HTS Bi-2223 composition. The RF signal reception and transmission of the HTS and professionally-made copper surface resonators were both accomplished by the inductively coupled approach (the bottom of Fig. 1 (d)) [9]. The professionally-made copper resonator was constructed using copper cable (Hitachi, Japan), which was 99.9999% (6N) purity copper, and a high-Q_s_ capacitor (22 pF and Q_s_ ≈ 1000 at 125 MHz, American Technical Ceramics, NY, USA). It was nonmagnetic and directly soldered at both ends. The diameter of the copper cable was 1.5 mm. The pick-up surface resonator with the diameter of 4 cm was placed below the HTS surface resonator and outside the cryostat in 300 K, approximately 5 mm from the HTS surface resonator. Furthermore, one trimmer capacitor (Voltronics Corp., NJ, USA) was soldered to the pick-up surface resonator. Matching, tuning and signal pick-up were done by adjusting the relative position of the signal pick-up surface resonator and tuning a variable trimmer capacitor to cancel out the imaginary part in Z_in_ [10]. The same geometry and position were used for both the HTS surface resonator and professionally-made copper surface resonator.

The glass-cryostat used for the phantom experiments was placed inside a gradient bore with a diameter of 12 cm. The separation between the HTS surface resonator and the phantom was about 5 mm. The cryostat with a vacuum jacket was designed to provide thermal insulation. The vacuum pressure was kept below 10^-7^ torr to reduce the thermal convention. The cryostat was constructed entirely from borosilicate glass (also called Pyrex glass) for thermal insulation. The coupled surface resonator was cooled to 77 K by liquid nitrogen in the patented-cryostat, which holds a temperature of 77 K for about 3 hours and could maintain the temperature until our LN_2_ supply was exhausted. The cryostat was placed on acrylic support and the phantom was placed under the cryostat. It should be noted that when the phantom was loaded under the HTS receiver in MRI, the frequency was shifted to a high frequency. During this time, adjusting the relative position of the signal pick-up surface resonator and tuning a variable trimmer capacitor can make the frequency to be reduced to 125.3 MHz. After reducing the temperature of the HTS coil with LN_2_ circulation, the frequency was also shifted to a high frequency. Then the tuning, matching, and signal pick-up needed to be performed again with the same method above.

Before the imaging experiment, the QF of the HTS and the professionally-made copper surface resonator were measured by the mutual coupling method using an HP 8751A Network Analyzer with the phantom (an 0.5 S.m-1 NaCl solution).

### SNR estimation

The Q_s_ is a highly crucial parameter when estimating the resistive loss of RF resonator. The frequency responses of the surface resonators and the values of Q_s_ were measured using a Hewlett Packard-8751A Network Analyzer set in the S_11_ (the reflection coefficient) mode. The S_11_ response is basically the ratio of the reflected power to the total transmitted power given by the network analyzer via the signal pick-up resonator [3]. The values of Q_s_ are then given by the ratio of the resonance frequency to the -3dB frequency bandwidth [11]. A study of the values of Q_s_ for Bi-2223-based LC resonator was reported in [1]. The Q_s_ of the resonant circuit are defined as *Q* = 2*ω*/Δ*ω*, where ω is the resonant frequency and Δω is the bandwidth of the resonant frequency at -3dB [12]. Assuming that the preamplifier noise voltage is negligible as compared to that of the resonator and sample, using equation (1) that the theoretical SNR gain of the HTS surface resonator at 77K over the professionally-made copper surface resonator at 300 K can be estimated [13,14].

$$\begin{array}{l} SNR^{gain}=\frac{SNR^{HTS}}{SNR^{copper}}=\sqrt{\frac{R_{cu}T_{cu}+R_{s}T_{s}}{R_{HTS}T_{HTS}+R_{s}T_{s}}}\\ =\sqrt{\frac{300Q_{U300(Cu)}^{-1}+300Q_{S}^{-1}}{77Q_{U77(HTS)}^{-1}+300Q_{S}^{-1}}} \end{array}$$ (1)

where SNR^HTS,77K^ and SNR^copper,300K^ are respectively the SNRs obtained from the HTS surface resonator at 77 K and the professionally-made copper resonator at 300 K; T_cu,300K_ and T_HTS,77K_ are the 300 K and 77 K, respectively; R_cu,300K_ and R_HTS,77K_ are the resistance of the professionally-made copper resonator at 300 K and the resistance of the HTS resonator at 77 K, respectively; Q_U300_(Cu) and Q_U77_(HTS) are the unloaded Q_s_ at 300 K and 77 K, respectively; and *Q*_*s*_ = *ωL*/*R*_*s*_ is the sample's Qs.

The RF signal reception and transmission of the HTS and professionally-made copper surface resonators in this study were both implemented by the inductively coupled approach. Inductive coupling has been used for several applications in magnetic resonance imaging (MRI), and it does cause small drop in SNR somewhat due to additional losses caused by the secondary coil [15]. Therefore, the loss due to inductive coupling should be considered. To measure the SNR loss due to inductive coupling, using equation (2) that the SNR loss of the HTS surface resonator at 77K over the professionally-made copper surface resonator at 300 K/77K can be estimated [16].

$$\delta =\frac{SNR_{c}}{SNR_{p}}=\sqrt{\frac{R_{in}-R_{s}}{R_{in}+R_{s}((T_{s}/T_{p})-1)}}$$ (2)

Where δ is the ratio of the SNR of the coupled coil system (SNRc) to the SNR of the primary coil alone (SNRp). R_in_ and R_s_ are the input resistance of the secondary coil coupled to the primary coil and the resistance of the secondary coil alone, respectively. T_s_ is the temperature of the secondary coil and T_p_ is the temperature of the primary coil, respectively.

We used the same pick-up resonator as the secondary coil, and it coupled inductively to the HTS surface resonator at 77K and the professionally-made copper surface resonator at 300K/77K, respectively. Input impedance of the pick-up resonator of both HTS and professionally-made copper surface resonators were measured using an HP 8751A Network Analyzer and also used as the indicator of the ratio of the SNR of the coupled coil system to the SNR of the primary coil alone.

### Imaging experiment

**Fig 2:**
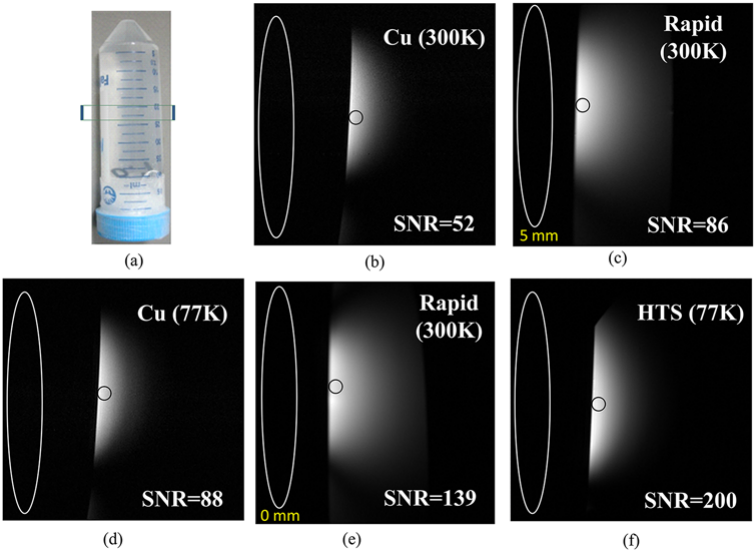
The imaging experiment of the phantom using the different coils. (a) A picture of the cylindrical phantom filled with 0.5 S.m^-1^ NaCl solution. (b) The copper surface resonator at 300 K with SNR of 52. (c) The QD surface resonator at 300 K with the distance of 5 mm with SNR of 86. (d) The copper surface resonator at 77 K with SNR of 88. (e) The QD surface resonator at 300 K close to the phantom with SNR of 139. (f) The HTS surface resonator at 77 K with SNR of 200. The SNR gain is mainly contributed by the reducing noise. A SNR gain of 1.43 was obtained by using the HTS-based surface resonator with the same acquisition time as the QD surface resonator close to the phantom.

To compare the different coils, phantom imaging was carried out. The cylindrical phantom was 29 mm in diameter and 90 mm in length and filled with a diluted NaCl solution to obtain a homogeneous conductivity of 0.5 S.m^-1^ (Fig. 2 (a)), which was close to that of biological tissues. The images were acquired using the fast spin echo sequence with TR/TE = 3500/62 ms, image matrix = 256 x 256, slice thickness = 1.24 mm and FOV = 6 x 6 cm^2^, and the in-plane resolution was 234 μm. The total scan time was 1 minute 36 seconds.

In a conventional image, the signal was measured by the mean value of pixels within the region of interest (ROI), with usual sizes of 0.5 cm by 0.5 cm. Standard deviation (STD) of background noise was measured using the largest possible ROI (avoid ghosting/aliasing or motion artifact regions). The SNR of an image was calculated as the ratio of the mean signal to the standard deviation of the background noise.

SNRs were calculated to compare the performance of the HTS surface resonator, the professionally-made copper surface resonator and the QD surface resonator. We used the mean value of pixels within the region of interest (ROI) to measure the signals of the phantom images obtained by the different coils. And the standard deviations (STDs) of the background noises were measured using the largest possible ROI which avoided ghosting/aliasing or motion artifact regions.

## Results

### Quality factor

The QFs of the HTS and the copper surface resonator were measured using the strategy described above. The unloaded and loaded QF of our 40 mm HTS surface resonator at 77 K were 1330 and 1050, respectively. Similarly, the unloaded and loaded QF of the professionally-made copper surface resonator at 300 K were 300 and 168, respectively. The unloaded and loaded QF of a professionally-made copper surface resonator at 77 K were 590 and 310, respectively. From equation(1), the predicated SNR gains with the HTS surface resonator at 77 K were 3.88 and 2.26 folds higher than that of the professionally-made copper surface resonator at 300 K and at 77 K, respectively.

### Inductive coupling efficiency

The SNR loss of the HTS surface resonator at 77K over the professionally-made copper surface resonator at 300 K/77K has been estimated. Due to the inductive coupling, the SNR degradations of the HTS surface resonator at 77K, the professionally-made copper surface resonator at 77K and the professionally-made copper surface resonator at 300K is approximately 10%, 15% and 18%, respectively.

### Phantom experiments

From the experimental images, the SNR of both images were calculated and the performance of the HTS surface resonator and the QD surface resonator were compared. In Fig. 2, the black circle represents the mean value of ROI, we chose the position at the sample which closest to the coil, and the diamater of the black circle was 0.5 cm. The white circle represented the STD in Fig. 2. At first, the distance between the QD surface resonator and the 0.5 S.m-1 NaCl phantom was 0 mm, the SNR was 139 (Fig. 2 (e)). However, when the distance between the QD surface resonator and the 0.5 S.m-1 NaCl phantom was increased to 5 mm, the SNR decreased to 86 (Fig. 2 (c)). The HTS surface resonator had to be placed inside the cryostat, thus the distance between the HTS surface resonator and the 0.5 S.m-1 NaCl phantom was 5 mm. The SNR using the HTS surface resonator at 77 K was 200 (Fig. 2 (f)). Afterwards, the HTS surface resonator was replaced with the professionally-made copper surface resonator inside the cryostat. The SNR using the professionally-made copper surface resonator at 77 K was 88 (Fig. 2 (d)) and the SNR using the professionally-made copper surface resonator at 300 K was 52 (Fig. 2 (b)). The SNR using the HTS surface resonator at 77 K was 200, 3.84-fold higher than the professionally-made copper resonator at 300 K, which was 52. Similarly, the SNR gain between the HTS surface resonator at 77 K over the QD surface resonator at 300 K is 1.43-fold.

### Simulation

**Fig 3:**
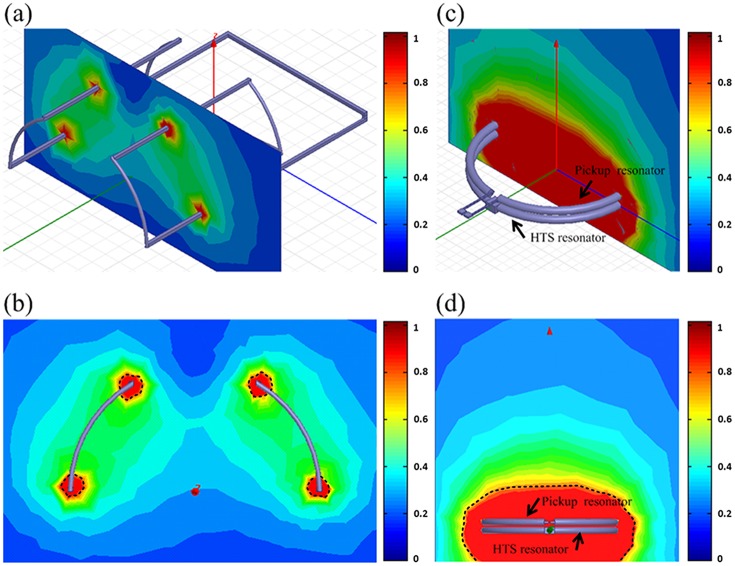
Ansoft HFSS simulated results of the QD surface resonator and the HTS surface resonators. (a) The 3D view of the QD surface resonator at 300K. (b) The X-axis view of the QD surface resonator at 300K. (c) The 3D view of the HTS surface resonators at 77K. (d) The X-axis view of the HTS surface resonators at 77K.

To compare the effect of the different geometry on the SNR, we simulated the magnetic field of the QD surface resonator at 300 K and the HTS surface resonator at 77 K. The relation between the normalized SNR and the different geometry was plotted according to the Ansoft HFSS simulation, and the simulated results of the QD surface resonator at 300 K and the HTS surface resonator at 77 K were shown in Fig. 3. Fig. 3 (a) and (b) represented the 3D and X-axis views of the QD surface resonator at 300 K, respectively. Similarly, the 3D and X-axis views of the HTS surface resonator at 77 K were shown in Fig. 3 (c) and (d), respectively.

In Fig. 3, the maximum value of the HTS surface resonator at 77 K was set to 1 and the normalized SNR of the QD surface resonator at 300 K was 0.43 approximately compared to the normalized SNR of the HTS surface resonator at 77 K. According to the results, the red region encircled by the dashed line represented the maximum value of the normalized SNR of the two coils. As for the QD surface resonator at 300 K, there were four red regions which were smaller than the red region of the HTS surface resonator at 77 K, as shown in Fig. 3 (b) and (d).

## Discussion

### SNR and imaging depth

**Fig 4:**
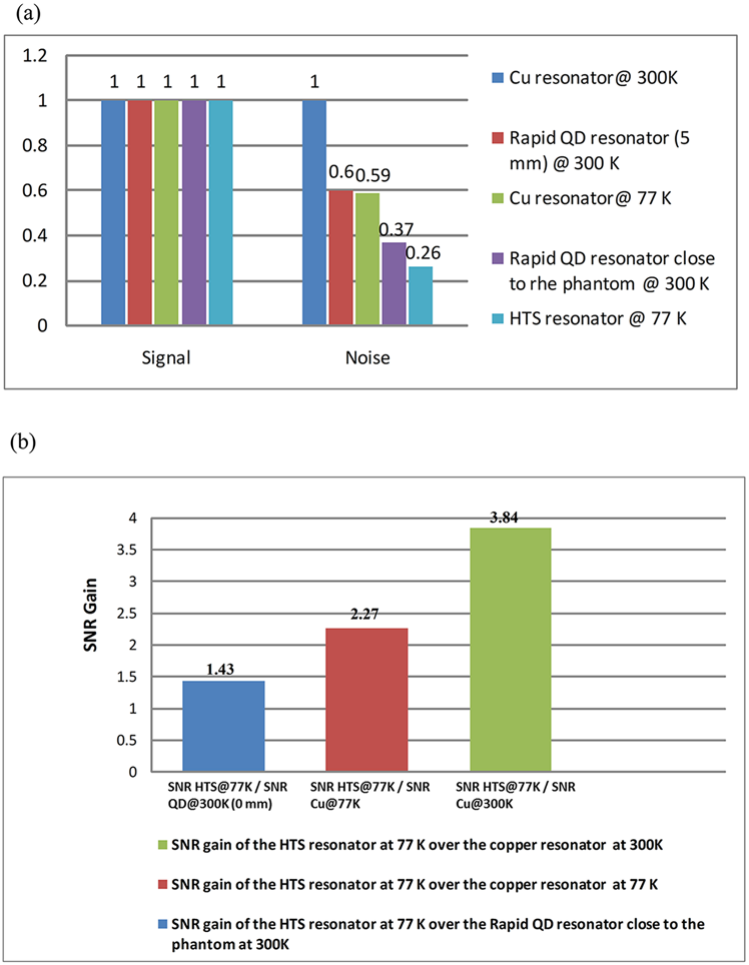
The comparison of SNR gain with the HTS surface resonator at 77 K, the copper surface resonator at 300 K, the copper surface resonator at 77 K, the QD surface resonator at 300 K with the distance of 5 mm and the QD surface resonator at 300 K close to the phantom. (a) It clearly shows almost no increase in signal intensity and mainly from the loss of noise. (b) The SNR gain of the HTS surface resonator at 77 K over the QD surface resonator at 300 K and professionally-made copper surface resonator at 77 K/ 300 K.

The distance between the professionally-made copper surface resonator and the phantom is d, the distance between the HTS surface resonator and the phantom is also d, while the distance between the QD surface resonator and the phantom is D. The magnitude of SNR using different kinds of surface resonators are listed in Table 2; the highest SNR is that of the HTS surface resonator at 77 K and the lowest SNR is that of the professionally-made copper surface resonator at 300 K. Although the diameter of the HTS surface resonator is larger than QD surface resonator, its SNR is still higher. The image results are used for evaluating the performance of the HTS surface ressonator at 77 K, the professionally-made copper surface resonator at 77 K and 300 K, and the commercial surface resonator at 300 K. The comparison of coronal phantom images from the HTS and professionally-made copper surface resonators are shown in Fig. 2. Images taken with the HTS surface resonator at 77 K have less background noise. The average SNR gain from measured data of the HTS surface resonator at 77 K is compared to that of the QD surface resonator and professionally-made copper surface resonators at 300 K, as shown in Fig. 4. The SNR gain is mainly from the noise reduction, since there is almost no increase in signal intensity, as shown in Fig. 4 (a). Fig. 4 (b) shows the SNR gain of the HTS surface resonator at 77 K over the QD surface resonator at 300 K and professionally-made copper surface resonator at 77 K/ 300 K.

**Table 2:** The SNR obtained from different kind of surface resonators.

Temperature (K)	d (mm)	The HTS surface resonator	The QD surface resonator	The copper surface resonator
T = 77 K	ds = 5 mm	SNR = 200		SNR = 88
T = 300 K	ds = 5 mm			SNR = 52
T = 300 K	D = 0 mm		SNR = 139	
T = 300 K	D = 5 mm		SNR = 86	

The distance between the HTS surface resonator/copper surface resonator and the phantom is d, while the distance between the QD surface resonator and the phantom is D.

The imaging depth with different surface resonators, can be seen in Fig. 2, the deepest imaging depth is observed with the QD surface resonator when the distance between the QD surface resonator and the phantom is 0 mm, while the thinnest imaging depth is observed with the professionally-made copper surface resonator at 300 K. Since the commercial resonator form is QD, it can cover a larger field-of-view (FOV) than the HTS and the professionally-made copper surface resonator. Therefore, the imaging depth of the QD surface resonator is deeper than the HTS and the professionally-made copper surface resonator.

For the signal-to-noise ratio (SNR) is proportional to B_1_, and according to Biot-Savart law, the profile of B_1_ field depends on the geometry of coils. It is well known that, as compared with the linear polarized of copper single loop surface coil, the SNR of the circular polarized of quadrature (QD) surface coil can improve SNR by a factor of root mean square 2 and reducing pulse power by half [17,18] at 300 K. When compared with the professionally-made copper coil at 300K, the QD surface resonator at 300 K demonstrates approximately a 40 percent reduction in noise ratio. On the other hand, the QD surface resonator can achieve and maintain an extended field of view (FOV), compared with the linear polarized of single loop surface coil. Nevertheless, what is remarkable in the HTS surface resonator at 77 K is that the SNR can be improved significantly even though the HTS surface resonator B_1_-sensitive region is smaller than that of the QD surface resonator at 300 K. Furthermore, we have confirmed the effect of the different geometry on the SNR between the QD surface resonator at 300 K and the HTS surface resonator at 77 K through Ansoft HFSS simulation. The Ansoft HFSS simulated results have been showed in Fig. 3. The different geometry of the two coils result in different normalized SNRs. As for the HTS surface resonator at 77 K, the normalized SNR is better when close to the coil, and with the increase of the distance, it will drop gradually, as shown in Fig. 3 (c) and (d). The similar situation has been shown in Fig. 3 (a) and (b) for the QD surface resonator at 300 K, but the rate of signal attenuation using the QD surface resonator at 300 K is larger than the HTS surface resonator at 77 K. Moreover, the HTS surface resonator at 77 K shows a higher intensity when compared with the QD surface resonator at 300 K. The results demonstrate that benefits can be expected by using the HTS surface coils at 77 K.

Although the field-of-view (FOV) and the penetration depth are limited due to the different geometry, the HTS surface resonator at 77 K improves the SNR of images obviously, and demonstrates approximately a 43% increase in SNR gain over the QD surface resonator at 300 K.

### HTS experiment with a patented-cryostat

In this study, the cryostat dimension is limited within a diameter of 12 cm to match the inserted animal gradient and the thermal insulation is mainly accomplished with the vacuum layer between the liquid nitrogen and imaging sample. The thermal insulation capability is approximately 1 hour for imaging *in-vivo* samples [3]. To improve the thermal insulation and prevent samples from freezing, it is important to choose adequate material for cryostat design.

On the issue of the SNR, the SNR of NMR is a function of the sample's distance from superconducting quantum interference device (SQUID). Using a theoretical calculation [19], the SNR equals 3 when the distance is 5 cm and is enhanced to 97 when the distance decreases to 1.5 mm. In this study, the HTS surface resonator is placed inside the vacuum jacket. The distance of the resonator-to-sample can be decreased to 5 mm, which is shorter than that reported in earlier studies [3,20,21]. Therefore, an enhanced SNR can be achieved. In addition, our cryostat has a vacuum jacket that provides good heat insulation to preserve liquid nitrogen for more than 3 hours [7]. The result reveals the stability of the cryostat with the blowing circulation system and shows promise for use in clinical scans. Looking at the hardware aspect, the imaging time can be significantly reduced by using parallel processing with phase array coils [22]. The potential benefits justify the development of practical HTS surface resonator for imaging systems despite considerable technical difficulties and challenges involved in the use of cryostat and volume coils with different shapes (such as solenoid, saddle or birdcage resonator).

This study demonstrates the advantages of using the 40 mm diameter HTS surface resonator over that of the 35 mm QD surface resonator on MRI researches in a 3T MRI. Our results show the cooled HTS surface resonator at 77 K providing a 1.43-fold SNR gain on phantom images over the QD surface resonator at 300 K. The results also show the cooled HTS resonator at 77 K providing a 3.84-fold SNR gain on phantom images over the professionally-made copper surface resonator at 300 K. The testing result agrees with the predicted one, and the difference between the predicted SNR gains and measured SNR gains is 1%.

Comapred with the QD surface resonator at 300K, the HTS surface resonator at 77K gains a much higher SNR, even though the geometries of the two coils are different. The HTS surface resonator at 77K can obtain a higher image quality, which has been showed in the phantom imaging results. The imaging time is also greatly reduced while maintaining the same image quality. Finally, a temperature stable HTS cryostat is presented in this study. Unlike previous studies [3,6,23,24,25], the distance of resonator-to-sample is improved to less than 5 mm. Thereore, the SNR is significantly enhanced compared with that reported in earlier papers [3,6,23,24,25].

With the HTS surface resonator, the SNR can be improved significantly, suggesting the HTS surface resonator as a potentially helpful diagnostic tool for MRI imaging in various applications. It is also suggested that some signals, which are previously obscured by noise, may now be revealed and further details of the image may be discovered.
